# Supplementary material for: De novo activated transcription of inserted foreign coding sequences is inheritable in the plant genome
Source: PLoS One. 2021 Jun 10;16(6):e0252674. doi: 10.1371/journal.pone.0252674 (PMC8191969; doi:10.1371/journal.pone.0252674)
Supplement: S1 Fig — (PDF) [file pone.0252674.s001.pdf]

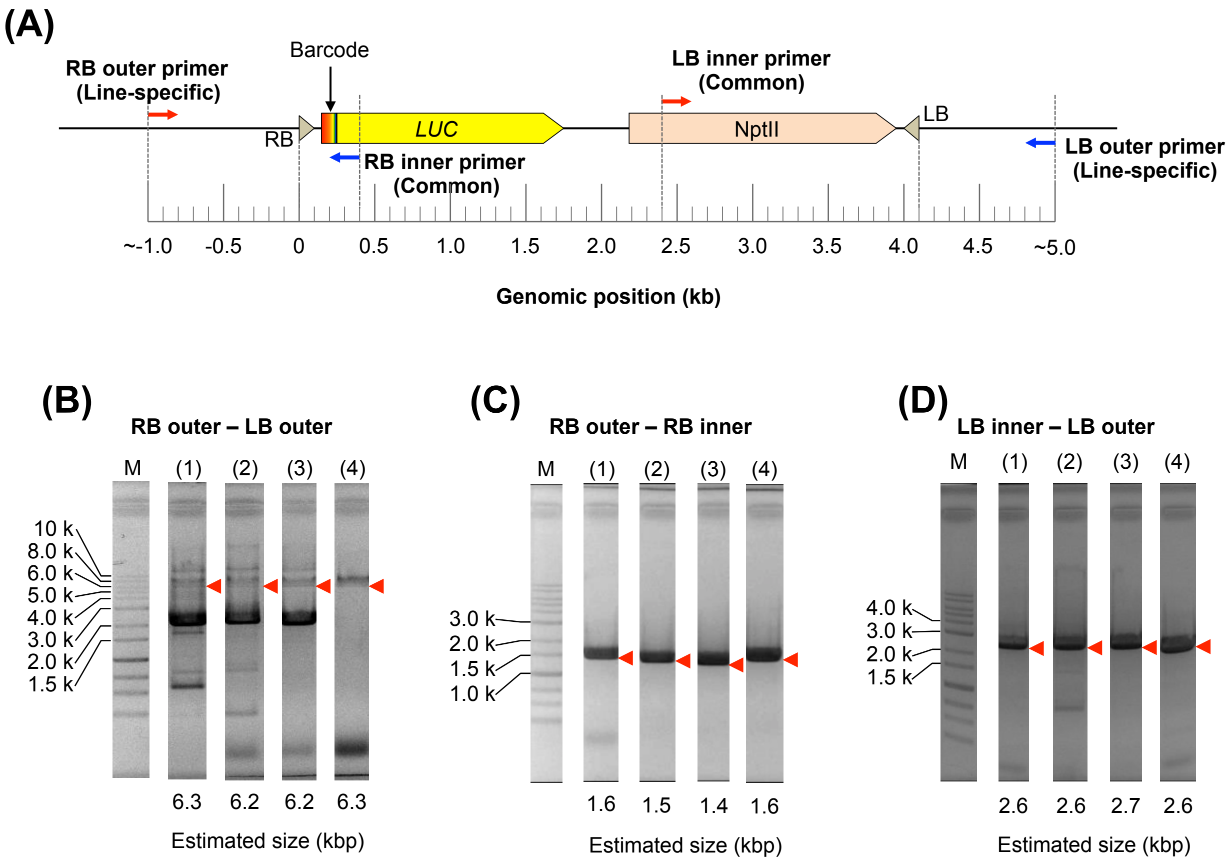

S1 Fig. Validation of *LUC* insertion loci.

(**A**) Schematic illustration of PCR-based validation of *LUC* insertion locus. In each selected transgenic line, RB outer and LB outer primers were designed about ±1.0 kb from RB and LB, respectively. RB inner and LB inner primers were in common with each line. (**B–D**) PCR products of randomly selected four lines were analyzed by the agarose gel electrophoresis. Primer sets used were (**B**) RB outer and LB outer, (**C**) RB outer and RB inner, and (**D**) LB inner and LB outer, respectively. The estimated sizes of PCR products in each line were calculated according to the determined locus by TRIP experiments. The bands corresponding to the expected sizes were indicated by red triangles. M: Molecular size marker.
