# Supplementary material for: De novo activated transcription of inserted foreign coding sequences is inheritable in the plant genome
Source: PLoS One. 2021 Jun 10;16(6):e0252674. doi: 10.1371/journal.pone.0252674 (PMC8191969; doi:10.1371/journal.pone.0252674)
Supplement: S2 Fig — (PDF) [file pone.0252674.s002.pdf]

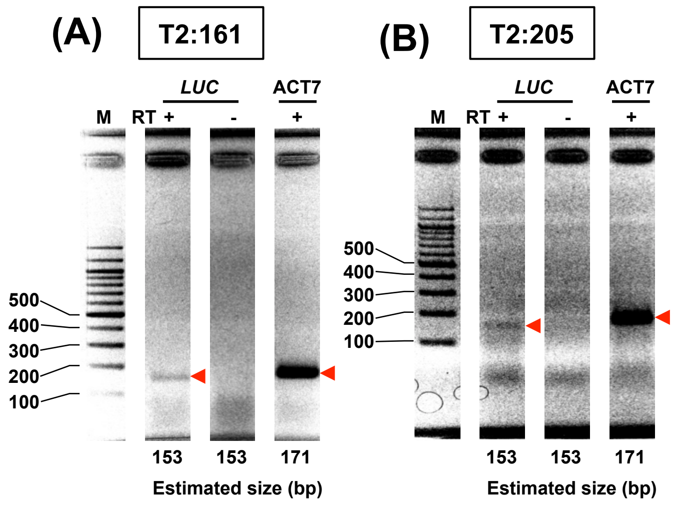

S2 Fig. Expression analysis of T2-plants.
(**A** and **B**) Expressions of (A) T2:161 line and (B) T2:205 line were validated by RT-PCR followed by gel electrophoresis. The bands corresponding to the expected sizes were indicated by red triangles. M: Molecular size marker, RT: Reverse transcription, and ACT7: AT5G09810.
