# Supplementary material for: De novo activated transcription of inserted foreign coding sequences is inheritable in the plant genome
Source: PLoS One. 2021 Jun 10;16(6):e0252674. doi: 10.1371/journal.pone.0252674 (PMC8191969; doi:10.1371/journal.pone.0252674)
Supplement: S1 Raw images — (PDF) [file pone.0252674.s006.pdf]

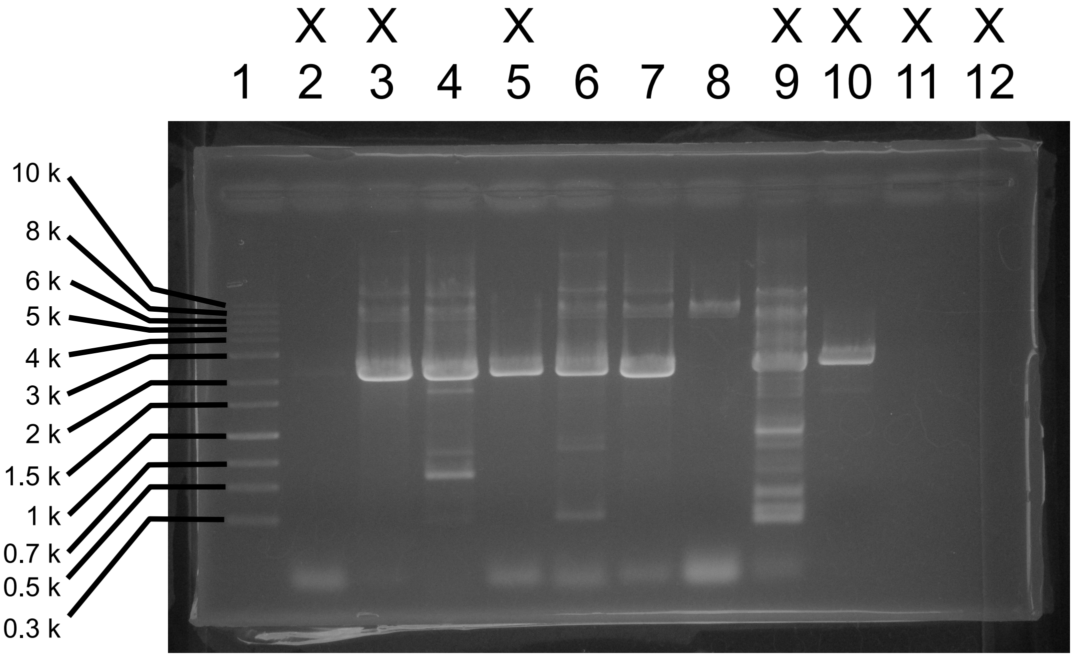

1. DNA molecular size marker (S1 Fig B, M)
2. T2:20, replicate1 (X)
3. T2:20, replicate2 (X)
4. T2:85 (S1 Fig B, (1))
5. T2:91 (X)
6. T2:161 (S1 Fig B, (2))
7. T2:201 (S1 Fig B, (3))
8. T2:205 (S1 Fig B, (4))
9. T2:245 (X)
10. T2:249 (X)
11. Blank (X)
12. Blank (X)

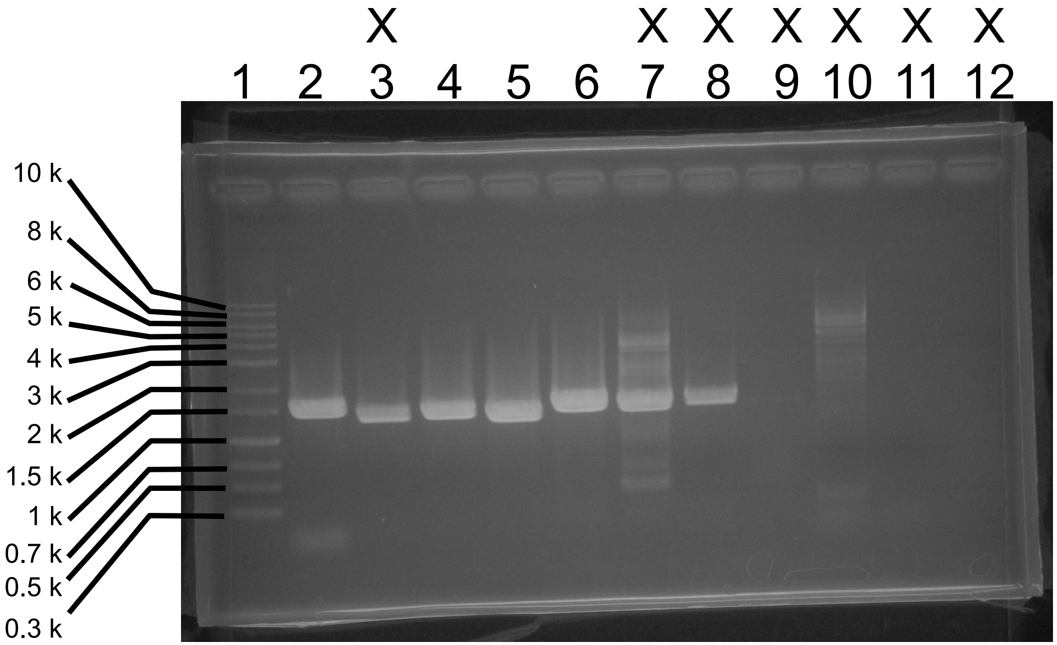

1. DNA molecular size marker (S1 Fig C, M)
2. T2:85 (S1 Fig C, (1))
3. T2:91 (X)
4. T2:161 (S1 Fig C, (2))
5. T2:201 (S1 Fig C, (3))
6. T2:205 (S1 Fig C, (4))
7. T2:245 (X)
8. T2:249 (X)
9. Blank (X)
10. T2:245 (X)
11. T2:249 (X)
12. Blank (X)

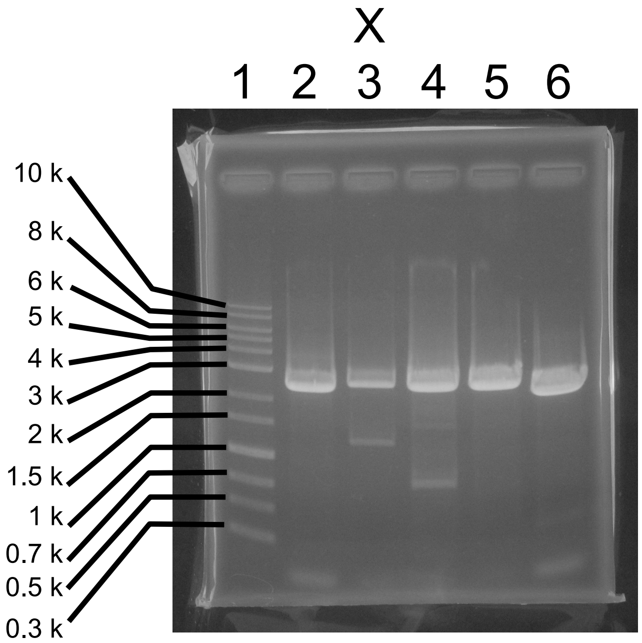

1. DNA molecular size marker (S1 Fig D, M)
2. T2:85 (S1 Fig D, (1))
3. T2:91 (X)
4. T2:161 (S1 Fig D, (2))
5. T2:201 (S1 Fig D, (3))
6. T2:205 (S1 Fig D, (4))

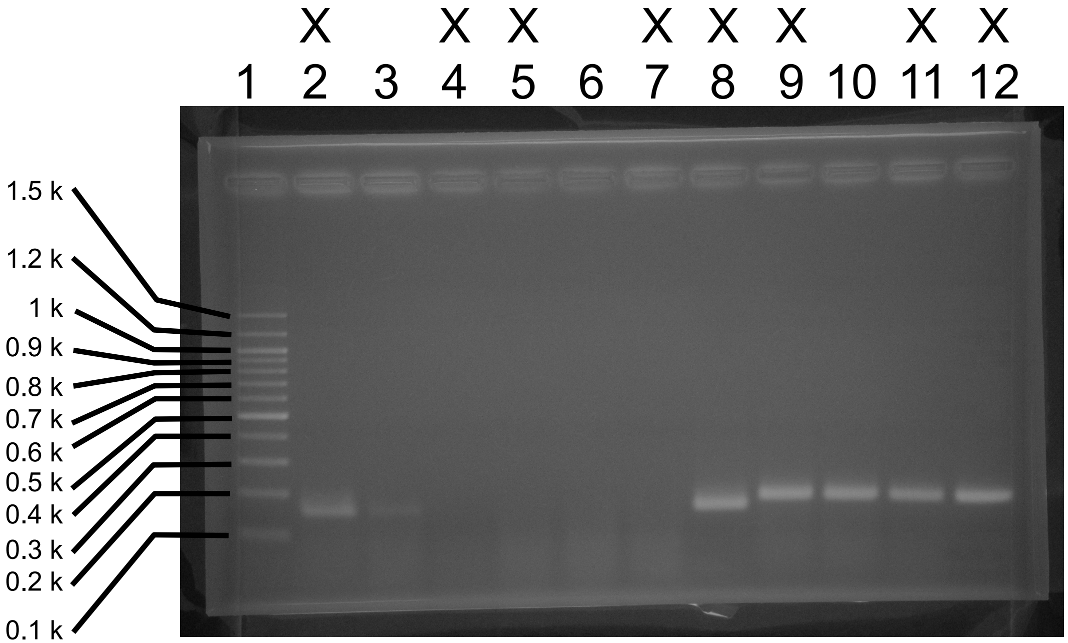

1. DNA molecular size marker (S2 Fig A, M)
2. T2:91 LUC RT+ (X)
3. T2:161 (S2 Fig A, LUC RT+)
4. WT LUC RT+ (X)
5. T2:91 LUC RT- (X)
6. T2:161 (S2 Fig A, LUC RT-)
7. WT LUC RT- (X)
8. T2:201 DNA LUC (X)
9. T2:91 ACT7 RT+ (X)
10. T2:161 (S2 Fig A, ACT7 RT+)
11. WT ACT7 RT+ (X)
12. T2:201 DNA ACT7 (X)

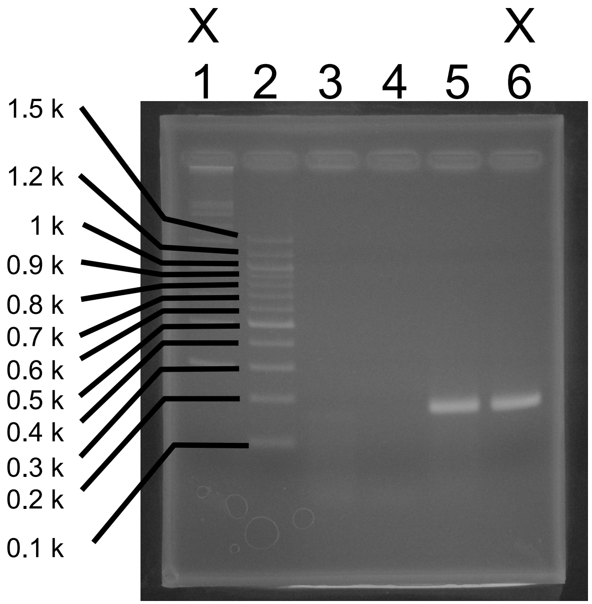

1. DNA molecular size marker (X)
2. DNA molecular size marker (S2 Fig B, M)
3. T2:205 (S2 Fig B, LUC, RT+)
4. T2:205 (S2 Fig B, LUC, RT-)
5. T2:205 (S2 Fig B, ACT7, RT+)
6. T2:205 ACT7, genomic DNA (X)
