# Supplementary material for: De novo activated transcription of inserted foreign coding sequences is inheritable in the plant genome
Source: PLoS One. 2021 Jun 10;16(6):e0252674. doi: 10.1371/journal.pone.0252674 (PMC8191969; doi:10.1371/journal.pone.0252674)
Supplement: S3 Fig — (PDF) [file pone.0252674.s003.pdf]

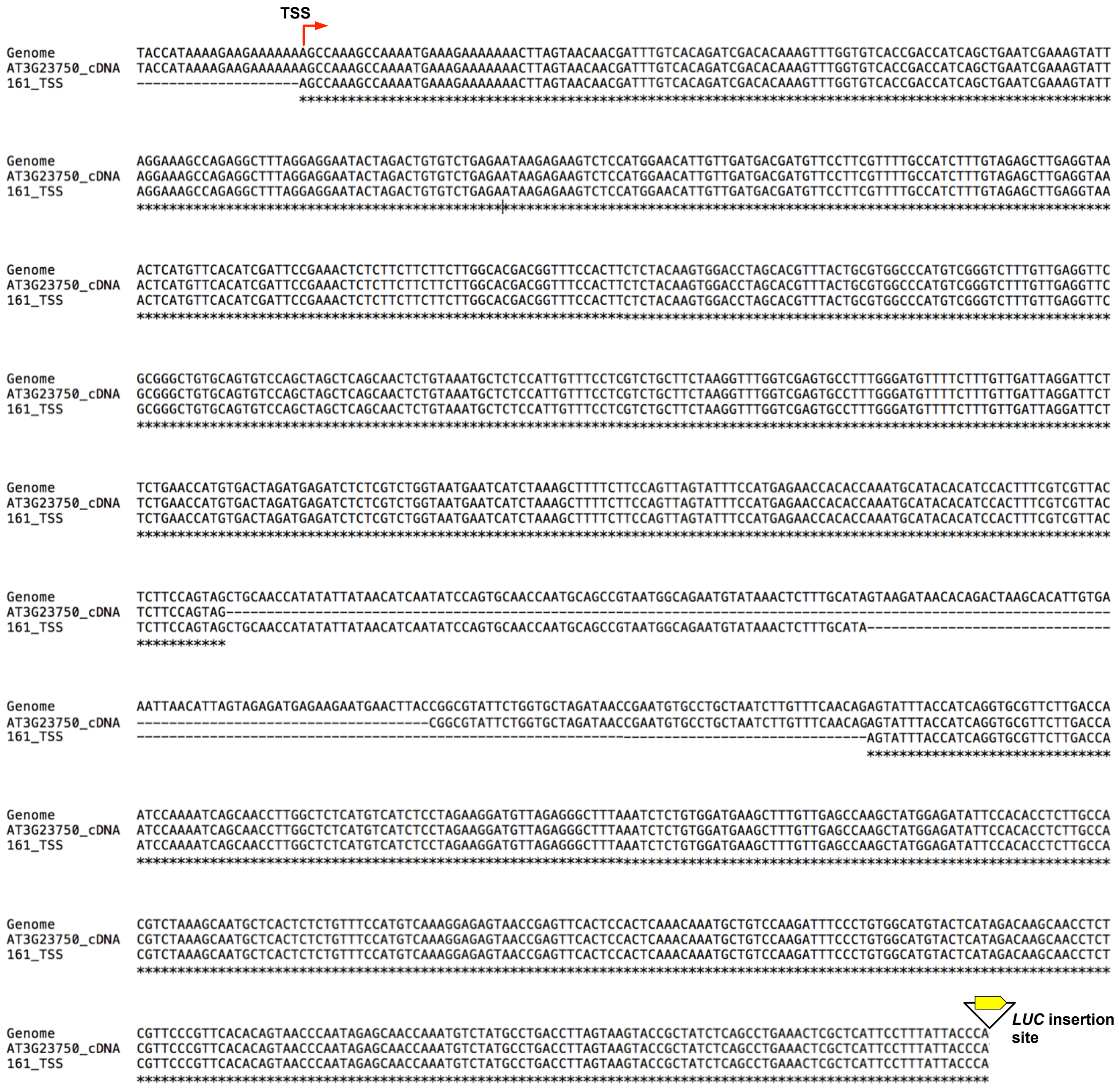

S3 Fig. Sequence alignment of TSS of T2:161 line.
Multiple alignments among genome, cDNA sequence of AT3G23750, and TSS flanking sequence of T2:161 line were showed. The red arrow indicated the TSS of the T2:161 line. Dash lines in the alignments indicated spliced regions. The alignment was calculated by using Mafft (v7.221) (Katoh and Standley, 2013).
